# Cephalometric Evaluation of the Effect of Complete Dentures on Retropharyngeal Space and Its Effect on Spirometric Values in Altered Vertical Dimension

**DOI:** 10.5402/2011/516969

**Published:** 2011-07-04

**Authors:** Prachi Gupta, Ram Thombare, A. J. Pakhan, Sameer Singhal

**Affiliations:** ^1^Department of Prosthodontics, Sharad Pawar Dental College, Datta Meghe Institute of Medical Sciences (Deemed University), Wardha 442004, Maharashtra, India; ^2^Department of Pulmonary Medicine, Jawaharlal Nehru Medical College, Datta Meghe Institute of Medical Sciences (Deemed University), Wardha 442004, Maharashtra, India

## Abstract

Role of complete dentures in reducing apnea-hypoapnea index in edentulous obstructive sleep apnea patient has shown promising results in previous studies. This study was undertaken to ascertain the role of complete denture and complete denture with slight increase in vertical dimension using custom made occlussal jig, on retropharyngeal space, posterior airway space, pharyngeal depth, and spirometric readings in comparison with those in edentulous group. Significant changes were observed in both intervention groups and thus, paving the way for doing further research for the consideration of using complete denture with modifications as an oral appliance in edentulous obstructive sleep apnea patient.

## 1. Introduction

Edentulism leads to decrease in size and tone of the pharyngeal musculature and is a crucial risk factor for obstructive sleep apnea (OSA) [1–3]. Literature review reveals that in a patient with obstructive sleep apnea, extraction of all teeth manifested worsening of the cardiorespiratory symptoms associated with almost doubling of the number of episodes of apnea/hypopnea per hour [3]. Obstructive sleep apnea syndrome (OSAS) is a disorder characterised by repeated obstruction of the upper airway, with consequent episodes of apnea and hypopnea during sleep, snoring, and daytime sleepiness [4, 5]. Increased pharyngeal collapsibility is reported to be a common cause of obstructive sleep apnea. It may be functional in nature, namely, muscular hypotonicity or anatomic in character due to conditions like macroglossia, retrognathia, micrognathiam, and soft tissue hyperplasia leading to reduction in size of the lumen of the airway. 

Loss of vertical dimension of occlusion which causes reduction of the lower face height and rotation of the mandible are some of the conditions which may lead to obstructive sleep apnea. In edentulous patients while recording lung function tests without dentures produces mild but significant decrease in inspiratory airflow rates [2], this may be suggestive of same threat to the patency of upper airway. Obstructive sleep apnea is a common disorder, especially in elderly people older than 50 years. About 61% of this group is estimated to meet the minimum criteria for obstructive sleep apnea [6]. Providing prosthodontic appliances, namely, mandibular repositioning devices and tongue repositioning devices may comply with the need as a treatment modality for these patients who present surgical risks or have had unsuccessful response to surgical procedures [4]. This study was undertaken to ascertain the role of complete dentures on modifications in the position of the jaw, tongue, soft tissues, and retropharyngeal space, thus precluding obstructive sleep apnea by restoring the lost vertical dimension and also to evaluate the effect of providing slight increase in vertical dimension of occlusion in same patient as well as to see the effect of same interventions on spirometric readings.

## 2. Materials and Methods

The 20 edentulous patients visiting the Department of Prosthodontics, complete denture prosthesis, were selected as subjects for this study. Following criteria of selection of the subjects were strictly adhered to: age group ranging between 40–70 years; healthy subjects from both genders with no systemic involvement especially respiratory diseases; residual alveolar ridge should be well formed/average; cooperative nature. All these patients were informed about the nature of study and the level of cooperation needed from them. After obtaining written consent, they were used for this study. The approval from Institutional Ethic Committee was obtained before the start of this study. The study was performed as follows.

**(I) Fabricating Complete Denture for Selected Subjects**

**Figure 1:**
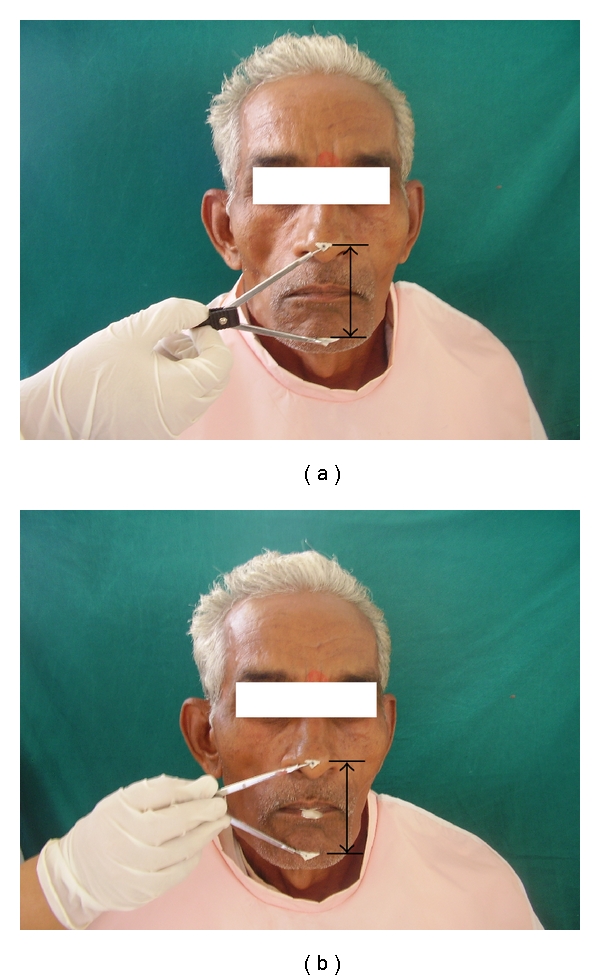
(a) Patient wearing complete denture with acceptable VDO. (b) Patient wearing complete denture with increased VDO by using acrylic JIG.

Routine procedure of impression making, recording jaw relation, selection, and arrangement of teeth was followed. After approval of try-in of waxed denture, they were processed following conventional method, using standard material for all subjects. Complete dentures so fabricated were provided proper vertical dimension of occlusion (Figure 1(a)).

**Figure 2:**
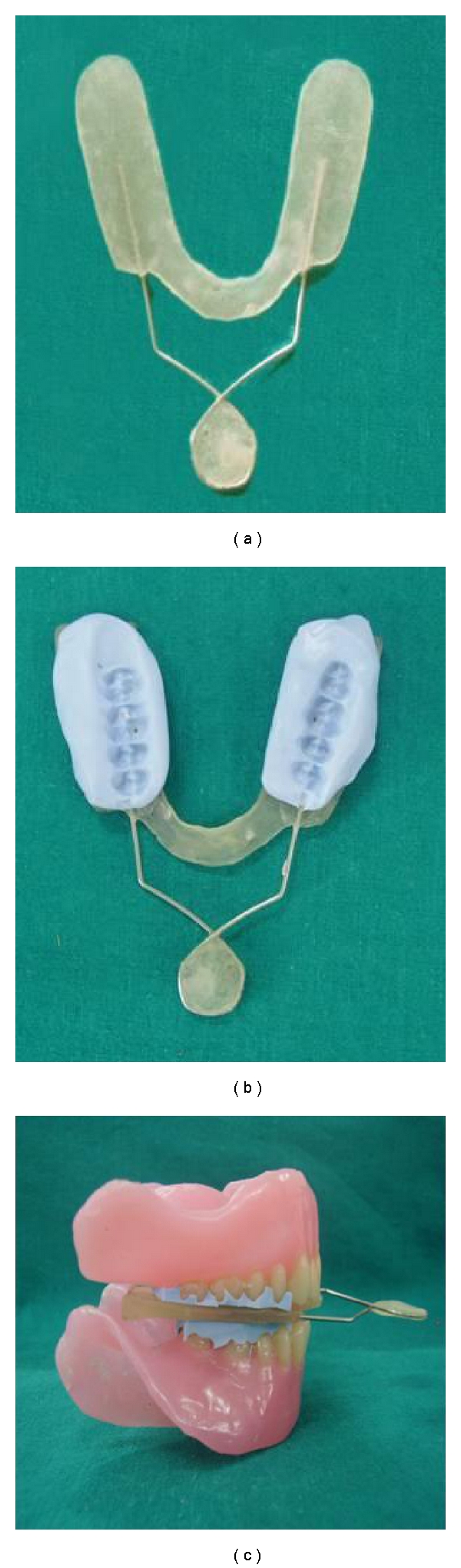
(a) Custom made acrylic occlusal JIG. (b) Occlusal surface of teeth registered in putty. (c) Increasing vertical dimension by using acrylic JIG.

**(II) Making of an Acrylic Occlusal JIG (Figure 2(a))**

For increasing vertical dimension of occlusion while shooting lateral cephalograph, an acrylic occlusal JIG was prepared using autopolymerising acrylic resin. About 2-3 mm thickness at posterior wings were provided to this acrylic JIG having wire loop as a handle.

**(III) Shooting Lateral Cephalographs**

Standardized lateral cephalographs were shooted using natural head posture (mirror technique) at end expiration, without swallowing and in centric occlusion. The source should be a minimum of 5 feet from the cassette. The centre X-ray beam was directed perpendicular to the right external auditory meatus. The time of exposure for an adult at this distance with medium speed cassette and medium speed film was 8/10 of a second at 70 KV and 12 mA value. Due precaution was taken during the procedure to avoid radiation hazards.

**Three lateral cephalographs for the same subject were shooted:**

**Figure 3:**
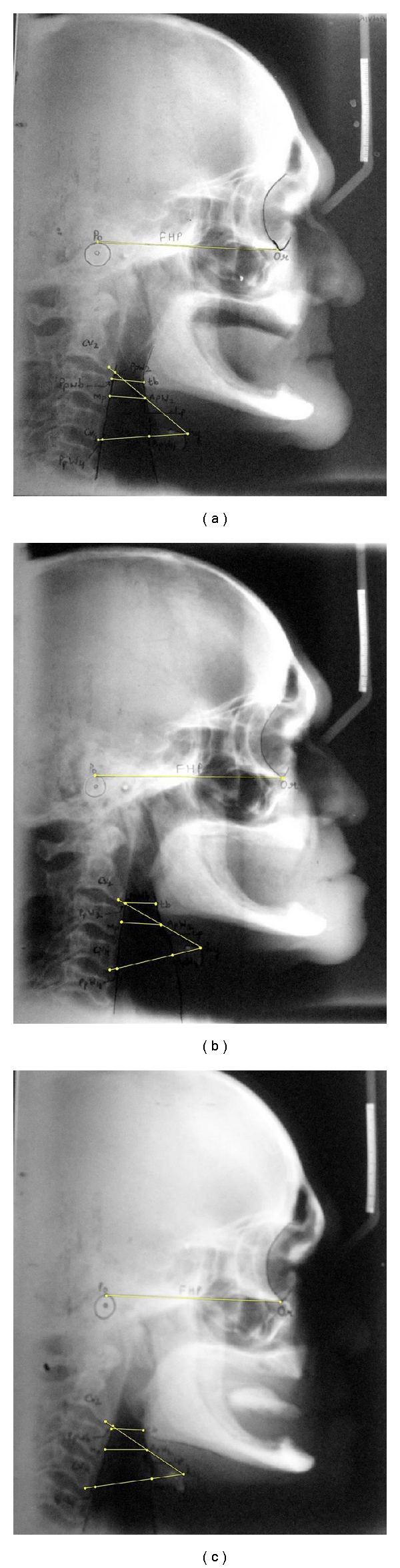
(a) Lateral cephalograph of the edentulous subject. (b) Lateral cephalograph of the same subject with acceptable VDO. (c) Lateral cephalograph of the same subject with increased VDO.

- lateral cephalograph of edentulous subjects (Figure 3(a)),
- lateral cephalograph (Figure 3(b)) of same edentulous subject wearing complete denture with acceptable vertical dimension of occlusion (Figure 1(a)),
- lateral cephalograph (Figure 3(c)) of same edentulous subject wearing complete denture having raised vertical dimension of occlusion using acrylic JIG with putty index (Figure 1(b)).

Occlusal JIG coated with putty on both sides was interposed between the upper and lower complete dentures in posterior region. Patient was advised to close the jaw for obtaining occlusal index of teeth in putty (Figure 2(b)). This will cause rise in vertical dimension of occlusion (Figures 2(c) and 1(b)). The lateral cephalograph was then shooted after hardening of putty index. This will stabilize both dentures while shooting cephalograph. Same procedure was followed for all 20 subjects used in this study. These cephalographs were then developed using standard technique.

**(IV) Tracing of Lateral Cephalograph**

**Figure 4:**
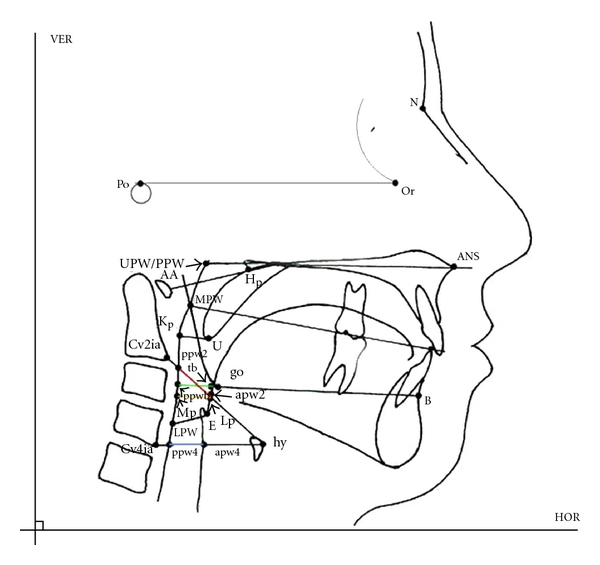
Cephalometric points used in the study.

An acetate tracing paper of proper size was affixed to the cephalograph with scotch tape. The tape was placed on the left side of the tracing paper. The cephalograph was then positioned on the X-ray illuminating table (X-ray viewer) so that the profile faces the right when it was viewed. A tracing was done following usual orthodontic practice [7]. A sharp, HB pencil and a plastic transparent 6 inches ruler was used for tracing (Figure 4).

**Following cephalometric reference points were identified:**

- Lp—point on anterior wall of oropharynx,
- Mp—point on posterior wall of oropharynx,
- cv2ia—the most anteroinferior point on the corpus of the second cervical vertebrae,
- cv4ia—the most anteroinferior point on the corpus of the fourth cervical vertebrae,
- ppw2—the posterior pharyngeal wall along the line intersecting Cv2ia and hy,
- ppw4—the posterior pharyngeal wall along the line intersecting Cv4ia and hy,
- hy—the most superior and anterior point on the body hyoid bone,
- apw2—the anterior pharyngeal wall along the line intersecting cv2ia and hy,
- apw4—the anterior pharyngeal wall along the line intersecting cv4ia and hy,
- tb—the intersection point of a line from point B through go and the base of the tongue,
- Point B—(supramentale)—the point at the deepest midline concavity on the mandibular symphysis between infradentale and pogonion,
- go—gonion—the constructed point of intersection of the ramus plane and the mandibular plane,
- Po—porion—the superior point of the external auditory meatus,
- Or—orbitale—the lowest point in the inferior margin of the orbit,
- ppwb—the intersection point of a line from B through go and the base of the posterior pharyngeal wall,
- Mp-Lp(Retropharyngeal space [RPS])—the smallest distance between the anterior (Lp) and posterior wall of oropharynx,
- apw2-ppw2—pharyngeal depth at level of second cervical,
- apw4-ppw4—pharyngeal depth at level of fourth cervical vertebrae.

PAS (posterior airway space) is linear distance between a point on the base of tounge (tb) and another point on the posterior pharyngeal wall (ppwb), both determined by an extension of line from point B through go.

Tracings were done for all subjects for all three groups of lateral cephalographs (Figures 3(a), 3(b), and 3(c)). Readings were obtained using digital caliper.

**(V) Spirometric Technique and Analysis**

**Figure 5:**
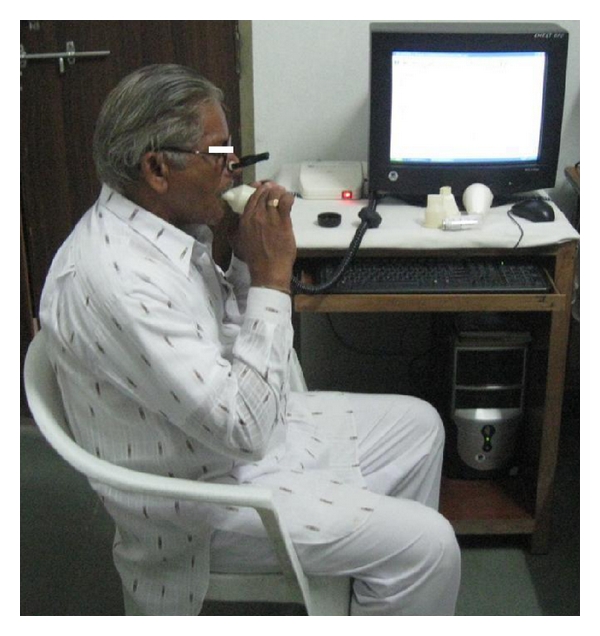
Patient performing spirometry.

Spirometry (Pulmonary Function Test) is a simple method of studying pulmonary ventilation by recording movements of air into and out of the lungs. Spirometry was done with spirometer with prior informed consent of patient (Figure 5). Acceptability criteria [8] included spirogram having good starts with extrapolated volume <5% of FVC or 0.15 Lt and satisfactory exhalation of 6 seconds or a plateau in volume-time curve. After three acceptable spirograms were recorded, reproducibility criteria were applied. The two largest FVC values within 0.2 Lt of each other and the two largest FEV1 values within 0.2 Lt of each other were taken. When both of these criteria were met, the session was concluded.

**The Test Was Performed**

1. for edentulous subjects (Without denture),
2. for same subjects with complete denture having acceptable vertical dimension of occlusion,
3. for same subjects with complete denture after increasing vertical dimension of occlusion by using occlusal JIG with putty index.

**Following Variables Were Taken into Consideration [8]**

1. FVC—It equals the amount of air that can be forcefully exhaled after complete inspiration.
2. FEV1—It equals the volume of air exhaled during the first second of expiration.
3. FEV1/FVC—Ratio is an invaluable indicator of respiratory disease and allows separation of ventilatory abnormalities into “restrictive” or “obstructive” patterns.
4. PIFR—It is peak inspiratory flow rate during inspiration and represents extrathoracic airways.

## 3. Results

The careful evaluation of the lateral cephalographs was carried out for all subjects used in this study, and the values so recorded (Table 1) were compared with edentulous subjects (control group) to know the effect on the retropharyngeal space, when complete dentures were used by same patients having acceptable vertical dimension of occlusion (first interventional group) and also by increasing vertical dimension of occlusion using acrylic JIG with putty index (second interventional group). Applying One-way ANOVA, Dunnett “*D*” test and unpaired “*t*” test, significant variations were found in retropharyngeal space, posterior airway space, pharyngeal depth at level of second cervical vertebrae and peak inspiratory flow rates between control and interventional groups and among first and second interventional groups, whereas no significant variations were seen in FVC, Fev1 and Fev1%. Pharyngeal depth at level of fourth cervical vertebrae showed significant variations only between control group and second interventional group (Table 2). 

## 4. Discussion

It is an established fact that edentulous patients tend to experience obstructive sleep apnea at a higher incidence than that of general population [9]. Loss or absence of teeth produces prominent anatomical changes that may influence upper airway size and function, such as loss of the vertical dimension of occlusion resulting into reduction of the lower face height and mandible rotation [10]. Rehabilitation of edentulous patient with complete dentures is an integral part of prosthodontic treatment modality. A denture not only provides esthetics and improves the phonetics but also restores the desired function of mastication and also provides adequate support to orofacial structures by restoring altered vertical dimension of face and also improves the conditions like OSA/hypopnea.

 Few studies have been done in past to investigate the role of complete denture in reducing apnea-hypopnea index in edentulous obstructive sleep apneic patients [1–3, 11]. Very few studies were carried out to evaluate the effect of wearing complete dentures in edentulous patients on spirometric readings [2].

These studies reported in literature have demonstrated that wearing complete dentures causes increase in the retropharyngeal space in supine position in edentulous patient with obstructive sleep apnea thereby reducing the severity of apnea-hypopnea events. The effect of complete denture fabricated with acceptable vertical dimension of occlusion and with increased vertical dimension of occlusion on retropharyngeal space and spirometric values in normal healthy edentulous patients has never been investigated in past. The present study was based on strong assumption that increasing the vertical dimension of occlusion by about 2-3 mm using custom made acrylic occlusal JIG would further result in increase in the retropharyngeal space. 

The subjects selected for this study were normal edentulous patients, and the fabrication of complete dentures using standard conventional method and routine materials provided a homogenous and representative sample. The clinical material used in study consisted of both male and female patients.

The present study demonstrated that significant changes were observed in retropharyngeal space with wearing of complete dentures fabricated with acceptable vertical dimension of occlusion (mean increase of 2.16 mm with “*P*” value <  0.05) in comparison to edentulous subjects. These changes were found to be more significant in same subjects after increasing vertical dimension of occlusion using custom made acrylic JIG (mean increase of 4.92 mm with “*P*” value <  0.05) in comparison to edentulous subjects. A similar study [1] carried out in past with 6 edentulous patients showed that removal of dentures lead to a striking decrease in retropharyngeal space from 15 mm to 6 mm, leading to increased severity of apnea-hypopnea events. Later on same results were obtained in another study [3] in which the authors hypothesized that edentulism might act in creating the apnoea condition by modifying anatomy and thereby affecting the functions of the pharyngeal airway and of tongue and may be by favouring inflammatory edema. Thus, they suggested that the advantage of removing dentures during sleep should be weighted against the risk of favouring upper airway collapse. 

It is also noticed in this study that significant changes were observed in posterior airway space, and pharyngeal depth at level of second cervical vertebrae in both the intervention groups; complete denture with acceptable vertical dimension of occlusion demonstrated mean increase of 1.88 mm in posterior airway space, and mean increase of 2.20 mm in pharyngeal depth in comparison to edentulous subjects with “*P*” value <0.05 and the complete denture after increasing vertical dimension of occlusion using custom made acrylic JIG of about 2-3 mm thickness, exhibited mean increase of 4.47 mm in posterior airway space and mean increase of 5.11 mm in pharyngeal depth at same level in comparison to edentulous subjects with “*P*” value <  0.05. 

In obstructive sleep apnea patients, there is an unusually small posterior airway space measurement and several bony structures surrounding the oropharynx which could be involved in the anatomic disarrangement leading to obstructive sleep apnea [12]. Meyer and Knudson in 1990 fabricated prosthesis to establish a vertical and protrusive jaw position in edentulous patients with obstructive sleep apnea and found that posterior airway space increased significantly with the prosthesis in edentulous patient [13]. Robertson CJ in 1998 theorized that increasing the vertical dimension of occlusion during fabrication of prosthesis for edentulous patient with obstructive sleep apnea was essential to ensure that dislodgement did not occur nocturnally [14]. Thus results are in agreement with the findings of this study.

In the latest study done in Japan, authors found that wearing complete dentures during sleep improves the apnea-hypopnea index in most of the patients. They further stated that this effect was due to reduction in partial pharyngeal obstruction when patient wore complete dentures during sleep [11].

Many researchers advocated that increase in posterior airway space could be achieved by advancing the mandible forward with or without the use of tongue retaining device and without increasing the vertical dimension of occlusion [15–18]. A mandibular advancement splint was used for edentulous obstructive sleep apnea patient using clinical procedures that were similar to those for fabricating a conventional complete denture without increasing the vertical dimension of occlusion. [16] A new functional appliance with combine characteristics of mandibular advancement splint and tongue retaining device with a custom made tongue tip housing without increasing vertical dimension of occlusion was fabricated for an edentulous obstructive sleep apnea patient popularly named as mandibular and tongue repositioner (MTR) [18]. 

An oral appliance fabricated like a denture with artificial teeth that not only reduced the severity of obstructive events but also provided an esthetic look to the patient with moderate obstructive sleep apnoea [17]. An implant retained mandibular repositioning appliance in the mandible was provided as a viable treatment modality in edentulous obstructive sleep apnea patients in another case report [19] documented in year 2007.

The advantage of using dentures in edentulous patient during sleep resulted in reducing apnea-hypopnea events in edentulous obstructive sleep apnea patient. This occurred due to the fact that wearing dentures induces modifications in the position of the jaw, tongue, soft tissue, and pharyngeal airway space [20] that may contribute to the reduction of apnea events. Moreover, since wearing complete dentures might not change the horizontal mandibular position as oral appliance do, it might help to restore the vertical mandibular position. Thus, the denture itself can act as an oral appliance and provides esthetic look to the patient. The result of this study is in confirmation of the above findings. Significant increase in retropharyngeal space (“*P*” value = 0.004) and posterior airway space (“*P*” value = 0.015) in “complete denture after increasing vertical dimension of occlusion” as compared to “complete denture with acceptable vertical dimension of occlusion” was observed. 

The disadvantages of wearing dentures during sleep are due to the fact that they are associated with chronic inflammatory changes, [21] leading to irritation and alveolar bone resorption in the denture-supporting area. In addition, increasing the vertical dimension of occlusion can cause strain on temporomandibular joint, and patient may need more time for adaptation to the same. 

The spirometric values were assessed with the wearing of complete denture with acceptable vertical dimension of occlusion and with increased vertical dimension of occlusion. It was observed that peak inspiratory flow rates (PIFR) were increased significantly in both intervention groups as compared with the values in edentulous state of the subjects. However, there were no significant changes in forced vital capacity, forced expiratory volume in 1 second, and FEV1% in intervention groups in comparison to edentulous subjects. This suggested that wearing dentures with acceptable vertical dimension of occlusion and increased vertical dimension of occlusion has significant effect on extrathoracic airways including retropharyngeal space. The increase in mean PIFR might be due to increase in retropharngeal space after wearing complete dentures, and results are in agreement with the findings of previous studies [2, 3]. 

Pellegrino et al. have also concluded that maximum inspiratory flow (PIF) is largely decreased with an extrathoracic airway obstruction, because the pressure surrounding the airways (which is almost equal to atmospheric) cannot oppose the negative intraluminal pressure generated with the inspiratory effort [22]. In contrast, it is little affected by an intrathoracic airway obstruction.

Thus, edentulous patients with obstructive sleep apnea may or may not use dentures during spirometric analysis of lung function test for assessment of intrathoracic airways (like for differentiating obstructive from restrictive lung diseases) but should always use dentures for assessment of extrathoracic airways (like in cases of obstructive sleep apnea patients, paratracheal tumours, paratracheal lymphadenopathy, and laryngeal inflammation). 

Therefore, this study can provide a breakthrough for further evaluation of the effect of increasing the vertical dimension of occlusion within acceptable limits in edentulous patients with obstructive sleep apnea and on spirometric parameters in future studies.

## 5. Conclusion

1. In edentulous subjects, the retropharyngeal space (RPS) and posterior airway space (PAS) were observed to be reduced. This was due to anatomical changes causing decrease in vertical dimension resulting into collapse of orofacial structures.
2. In same edentulous subjects, after wearing complete dentures having acceptable VDO, the retropharyngeal space (RPS) and posterior airway space (PAS) were found to be increased which was due to restored vertical dimension of occlusion.
3. When same edentulous subjects wearing complete denture after increasing vertical dimension of occlusion was subjected to analysis of these values (RPS & PAS), it was observed that there was marked increase in these values more than the values observed in same subjects wearing complete dentures with acceptable vertical dimension of occlusion.
4. Peak inspiratory flow rate was observed to be increased in subjects wearing complete dentures with acceptable vertical dimension of occlusion as well as increased vertical dimension of occlusion. This increase in PIFR was slight on higher side in subjects wearing complete denture with increased VDO as compared to acceptable VDO.
5. These significant differences are important from the point of view of edentulous patients having obstructive sleep apnea in which there are unusually small retropharyngeal and posterior airway spaces. Providing complete dentures fabricated with acceptable and may be with increased vertical dimension of occlusion within limits of acceptability of tissues to these patients can minimize the pharyngeal collapsibility, thereby reducing apnea-hypopnea events.
6. Further research is needed with edentulous patients with OSA to explore the possibility of using modified complete dentures or providing permissible adjustments to increase vertical dimension of occlusion of complete denture for using the same as an oral appliance in OSA patients to evaluate the effect of using such modified dentures on orofacial structures for providing comfort to such individuals.

## Figures and Tables

**Table 1:** Recorded lateral cephalograph and spirometric values.

		Range (Min.–Max. value)	Mean value	Percentage increase in comparison to control group
Retropharyngeal space (Mp-Lp) in mm	Edentulous subjects (control group)	6.59–20.18	11.97	0%
	First interventional group	9.64–22.32	14.14	18.12%
	Second interventional group	12.73–22.78	16.90	41.18%

Posterior airway space in mm	Edentulous subjects (control group)	6.08–23.07	12.63	0%
	First interventional group	7.98–24.96	14.50	14.80%
	Second interventional group	12.59–26.06	17.10	35.39%

apw2-ppw2 in mm	Edentulous subjects (control group)	8.98–22.15	13.55	0%
	First interventional group	9.92–24.39	15.75	16.23%
	Second interventional group	14.95–25.48	18.67	37.78%

apw4-ppw4 in mm	Edentulous subjects (control group)	10.56–26.44	18.74	0%
	First interventional group	12.56–27.07	20.33	8.48%
	Second interventional group	14.08–31.97	22.01	17.44%

FVC in % predicted	Edentulous subjects (control group)	54–98	76.75	0%
	First interventional group	63–98	77.35	0.78%
	Second interventional group	60–97	77.95	1.56%

Fev1 in % predicted	Edentulous subjects (control group)	54–101	76.30	0%
	First interventional group	48–103	77.40	1.44%
	Second interventional group	44–91	77.15	1.11%

Fev1% in % predicted	Edentulous subjects (control group)	59–114	94.40	0%
	First interventional group	62–111	94.80	0.42%
	Second interventional group	64–111	94.80	0.42%

PIFR in L/sec	Edentulous subjects (control group)	1.43–3.75	2.39	0%
	First interventional group	1.51–4.84	2.93	22.59%
	Second interventional group	1.54–5.24	3.29	37.65%

**Table 2:** Statistical analysis of cephalograph and spirometric values.

	One-way ANOVA (*P* value)	Dunnett “*D*” test A = *P* value between control group and first interventional group B = *P* value between control group and second interventional group	Unpaired “*t*” test (*P* value)
Retropharyngeal space	0.000 *S	A = 0.007 S B = 0.000 S	0.004 S
Posterior airway space	0.000 S	A = 0.042 S B = 0.000 S	0.015 S
apw2-ppw2	0.000 S	A = 0.016 S B = 0.000 S	0.002 S
apw4-ppw4	0.001 S	A = 0.103 NS B = 0.000 S	0.099 NS
FVC	0.335 **NS	A = 0.862 NS B = 0.628 NS	0.769 NS
Fev1	0.775 NS	A = 0.762 NS B = 0.760 NS	0.998 NS
Fev1%	0.889 NS	A = 0.921 NS B = 0.853 NS	0.914 NS
PIFR	0.000 S	A = 0.002 S B = 0.000 S	0.031 S

*S = significant.

**NS = non significant.

## References

[1] Bucca C, Carossa S, Pivetti S, Gai V, Rolla G, Preti G (1999). Edentulism and worsening of obstructive sleep apnoea. *The Lancet*.

[2] Bucca CB, Carossa S, Colagrande P (2001). Effect of edentulism on spirometric tests. *American Journal of Respiratory and Critical Care Medicine*.

[3] Bucca C, Cicolin A, Brussino L (2006). Tooth loss and obstructive sleep apnoea. *Respiratory Research*.

[4] Ivanhoe JR, Cibirka RM, Lefebvre CA, Parr GR (1999). Dental considerations in upper airway sleep disorders: a review of the literature. *The Journal of Prosthetic Dentistry*.

[5] Magliocca KR, Helman JI (2005). Obstructive sleep apnea: diagnosis, medical management and dental implications. *Journal of the American Dental Association*.

[6] Flemons WW, Buysse D, Redline S (1999). Sleep-related breathing disorders in adults: recommendations for syndrome definition and measurement techniques in clinical research. *Sleep*.

[7] Athanasiou EA, Papadopoulos M, Lagoudakis M, Goumas P, Athanasiou EA (1995). Assesment of pharyngeal relationships. *Orthodontic Cephalometry*.

[8] American Thoracic Society (1991). Lung function testing: selection of reference values and interpretative strategies. *The American Review of Respiratory Disease*.

[9] Ancoli-Israel S, Gehrman P, Kripke DF (2001). Long-term follow-up of sleep disordered breathing in older adults. *Sleep Medicine*.

[10] Douglass JB, Meader L, Kaplan A, Ellinger CW (1993). Cephalometric evaluation of the changes in patients wearing complete dentures: a 20-year study. *The Journal of Prosthetic Dentistry*.

[11] Arisaka H, Sakuraba S, Tamaki K, Watanabe T, Takeda J, Yoshida KI (2009). Effects of wearing complete dentures during sleep on the apnea-hypopnea index. *International Journal of Prosthodontics*.

[12] Guilleminault C, Riley R, Powell N (1984). Obstructive sleep apnea and abnormal cephalometric measurements. Implications for treatment. *Chest*.

[13] Meyer JB, Knudson RC (1990). Fabrication of a prosthesis to prevent sleep apnea in edentulous patients. *The Journal of Prosthetic Dentistry*.

[14] Robertson CJ (1998). Treatment of obstructive sleep apnoea in edentulous patients—design of a combination appliance: a case study. *The New Zealand Dental Journal*.

[15] Smith AM, Battagel JM (2004). Non-apneic snoring and the orthodontist: radiographic pharyngeal dimension changes with supine posture and mandibular protrusion. *Journal of Orthodontics*.

[16] Nayar S, Knox J (2005). Management of obstructive sleep apnea in an edentulous patient with a mandibular advancement splint: a clinical report. *Journal of Prosthetic Dentistry*.

[17] Taner T, Aydinatay BS, Turkyilmaz I, Demir AU (2007). The use of modified mandibular advancement device in the treatment of a partially edentulous patient with obstructive sleep apnea. *Turkey Dental Journal*.

[18] Kurtulmus H, Cotert SH (2009). Management of obstructive sleep apnea in an edentulous patient with a combination of mandibular advancement splint and tongue-retaining device: a clinical report. *Sleep and Breathing*.

[19] Hoekema A, De Vries F, Heydenrijk K, Stegenga B (2007). Implant-retained oral appliances: a novel treatment for edentulous patients with obstructive sleep apnea-hypopnea syndrome. *Clinical Oral Implants Research*.

[20] Erovigni F, Graziano A, Ceruti P, Gassino G, De Lillo A, Carossa S (2005). Cephalometric evaluation of the upper airway in patients with complete dentures. *Minerva Stomatologica*.

[21] Marcus PA, Joshi A, Jones JA, Morgano SM (1996). Complete edentulism and denture use for elders in New England. *Journal of Prosthetic Dentistry*.

[22] Pellegrino R, Viegi G, Brusasco V (2005). Interpretative strategies for lung function tests. *European Respiratory Journal*.

